# The Path Is the Goal: How Transformational Leaders Enhance Followers’ Job Attitudes and Proactive Behavior

**DOI:** 10.3389/fpsyg.2018.02338

**Published:** 2018-11-29

**Authors:** Barbara Steinmann, Hannah J. P. Klug, Günter W. Maier

**Affiliations:** Work and Organizational Psychology, Department of Psychology, Bielefeld University, Bielefeld, Germany

**Keywords:** transformational leadership, goal setting, organizational goals, goal importance, goal attainability

## Abstract

While leading through goals is usually associated with a task-oriented leadership style, the present work links goal setting to transformational leadership. An online survey with two time points was conducted with employees to investigate the influence of transformational leadership on followers’ job satisfaction, organizational commitment, and proactive behavior via goal attributes. Findings indicate that transformational leaders influence the extent to which followers evaluate organizational goals as important and perceive them as attainable. Multiple mediation analysis revealed that these goal attributes transmit the effect of transformational leadership on followers’ job attitudes and proactive behavior. However, goal importance and goal attainability seem to be of differential importance for the different outcomes.

## Introduction

Although the setting of goals has been emphasized to be one of the most important tasks of leaders (e.g., Tett et al., 2000), goals and leadership have commonly been considered from two relatively independent research perspectives (cf. Berson et al., 2015). In the field of goal research many efforts centered on the setting of goals in organizational contexts. As a core finding, a multitude of studies (for an overview: Locke and Latham, 2002) revealed that setting specific and moderately difficult goals results in increases of an individual’s performance as such goals direct one’s attention, induce greater effort, enhance one’s persistence, and elicit the use of task-related knowledge and strategies (Locke and Latham, 2002). Studies further showed that the strength of this association depends on certain goal attributes, an individual’s self-efficacy beliefs, as well as feedback on and the complexity of the task. Apart from its impact on an individual’s job performance and work motivation, goal setting is also an important determinant of one’s self-regulation (Latham and Locke, 1991). Their self-regulative function results as specific and difficult goals point out a discrepancy between a current and a future state and clarify the acceptable level of performance (Latham and Locke, 1991). Goals, however, may not only be set by another person but also by an individual him-/herself. Personal goals and their pursuit have been another line of interest for goal researchers (e.g., Emmons, 1986; Brunstein, 1993). In the field of leadership research, goals have initially been assigned a dominant role in those conceptions, which highlight a leader’s task orientation. Task-oriented leaders focus on getting their work done and completing assignments (Bass, 1990). Such leaders therefore emphasize goals, foster their achievement, and monitor followers’ goal pursuit. In this regard, goals may be seen as a means to exert control in leader-follower interactions.

Instead of viewing the assignment of goals as a way to monitor followers, in the present study, we embed the goal setting of leaders into the context of motivating and enabling subordinates. In so doing, we concentrate on the construct of transformational leadership, as transformational leaders (TLs) not only have high performance expectations (Bass, 1985), but rather inspire and empower their subordinates (Bass and Riggio, 2006). In motivating and enabling followers, goals have variously been assigned a central role in the theory of transformational leadership (e.g., Shamir et al., 1993; Conger and Kanungo, 1998). Therefore, a goal-perspective to transformational leadership is straightforward.

Given that setting goals is a common leadership task (Tett et al., 2000), it is indispensable to incorporate well-founded knowledge accumulated in the field of goal research into study efforts on effective leadership. Only if we consider both research domains jointly, we can get the best picture possible of how leaders influence followers and the way they pursue the goals these leaders set. Intertwining findings and theoretical assumptions on goal setting, self-regulative goal pursuit, and personal goals with empirical evidence and theorizing on transformational leadership, we assume TLs to foster followers’ perception of organizational goals to be important and attainable, and by these means, to increase their job satisfaction, organizational commitment, and proactive behavior. That way, the present study helps in bringing together the different streams of research and to generalize extant evidence on assigned and personal goals to the goal setting within leader-follower-interactions. In so doing, our study investigates fundamental assumptions on the inner workings of transformational leadership for which empirical evidence is yet scarce. As such, the present work also contributes to further substantiating theoretically derived mechanisms of transformational leadership and thus to our understanding of how these leaders exert their extraordinary influence on followers.

### Motivating and Enabling Employees: The Transformational Leadership Approach

TLs motivate followers to commit themselves to organizational objectives and to realize performance outcomes, which exceed beyond expectations. According to Bass (1985), leaders accomplish this process of motivating and transforming followers by (1) heightening their awareness of the importance and value of designated goals, (2) encouraging them to transcend self-interests for the good of the organization or team, and (3) activating their higher order needs as TLs articulate an inspiring vision and act as role models in attaining the vision. More specifically, TLs are able to ideally influence subordinates due to their exceptional charisma and prompt followers to personally identify with them (Bass, 1985). Based on this emotional attachment, TLs instill within followers the desire to emulate their leaders and thus become followers’ role models. TLs envision an appealing future goal state for their team or the entire organization and express confidence in followers’ abilities to attain this higher-order goal (Bass, 1985). By this means, they inspirationally motivate followers to achieve more than expected. As they tie the ideological vision to the collective’s future, TLs foster the acceptance of group goals and enhance the cooperation within teams (Podsakoff et al., 1990). Besides, they intellectually stimulate followers to question their way of working and to take on new perspectives increasing subordinates’ awareness of problems that way (Podsakoff et al., 1990). TLs clearly express the high performance demands they have and expect excellence and high quality work from followers (Podsakoff et al., 1990). Concurrently, they also attend to followers’ needs, listen to their particular concerns, and are individually considerate toward them (Bass, 1985).

After the key behaviors used to transform and motivate followers had been identified, Conger and Kanungo (1998) claimed that more insights into the process of motivating and transforming followers were needed and called for a more processual perspective on transformational leadership. They developed a three-stage model, which aimed at illustrating how TLs transform subordinates and move them from an existing present state toward some future state. According to this model, TLs first examine the current situation at work and its surrounding environment. In this initial stage, they actively search the status quo for existing or potential shortcomings. Based on the deficiencies they identify, goals are then derived, formulated, and conveyed in the second stage. By articulating a very discrepant and idealized goal, TLs provide a sense of challenge and a motivating force for change to their followers (Conger, 1999). In the final stage, they build trust in the goals they disseminate and demonstrate how these goals can be attained. The model thus highlights the communication and implementation of a vision or goal as a key mechanism of transformational leadership.

### Goal Setting, Self-Regulation, and Personal Goals

In the work context, goals may help to predict, explicate, and affect an employee’s job performance (Locke and Latham, 2002). By setting followers’ goals, leaders create a discrepancy between a current situation and a future state and, with regard to work-related tasks, emphasize what constitutes an adequate level of performance. That way, they provide a sense of purpose, which coordinates and guides their followers’ action (Latham and Locke, 1991).

After a goal is communicated or set, leaders often do not have direct control over their subordinates’ goal pursuit anymore and followers have to plan and organize the goal striving process autonomously. In order to attain organizational goals, employees therefore have to be able to self-regulate at work. Traditionally, self-regulation is defined as processes that “enable an individual to guide his/her goal-directed activities over time and across changing circumstances (contexts), [… including the] modulation of thought, affect, behavior, or attention” (Karoly, 1993, p. 25). This definition points out that in the process of self-regulation, goals are an essential component (Vancouver, 2000). Moreover, it describes self-regulation as a volitional process of translating the goals, which have been set into action. In a series of experiments, Oettingen et al. (2001) identified three self-regulatory thought processes, which are of relevance within an autonomous goal setting process: mentally contrasting the desired future with reality, dwelling on negative aspects of the current reality, and indulging in the desired future. The authors observed that as a function of these three self-regulatory thoughts, feelings of identification with the goal, expectations of success, and effortful goal striving result.

Self-regulated goal striving is also addressed in the field of *personal goal* research. Personal goals are set by an individual him-/herself and are therefore person-specific. Models of personal goal pursuit emphasize the personal significance and uniqueness of these goals and acknowledge the autonomy and self-determination during the goal striving process (e.g., Emmons, 1986; Brunstein, 1993). Knowledge gathered in the domain of personal goals may give valuable insights into the way TLs facilitate their followers’ goal pursuit. As TLs intertwine the goals they set with followers’ self-concepts (Shamir et al., 1993) and lead them to internalize these goals (Bono and Judge, 2003), subordinates perceive these goals to be highly self-consistent (Shamir et al., 1993) and feel goal-directed actions to be driven by personally held values (Bono and Judge, 2003). TLs hence seem to be able to turn organizational goals into followers’ personal goals. According to the personal goal model of well-being (for an overview: Brunstein et al., 1999), which is well-established in the field of personal goal research, there are two decisive factors that determine one’s success in pursuing personal goals as well as the subjective well-being of the goal striver: the valence followers attach to the goals and the degree to which they perceive the goals to be attainable. Whereas a goal’s importance increases one’s determination in pursuing the goals (Maier and Brunstein, 2001), the evaluation of a goal to be attainable first leads individuals to decide to pursue that goal (Heckhausen and Kuhl, 1985). Maier and Brunstein (2001) adapted this model to the work domain and report evidence, which suggests that the two goal attributes account for changes in job satisfaction and organizational commitment. They conclude that “to achieve well-being and avoid distress, it is important for individuals to have both a strong sense of commitment to valued goals and a life situation that provides favorable conditions to materialize these goals” (Maier and Brunstein, 2001, p. 1035).

Combining self-regulation theory and the personal goal model, one can assume that the goal attributes highlighted in the personal goal model result from the self-regulatory processes Oettingen et al. (2001) found to be related to an autonomous goal striving. Goal importance and goal attainability may thus be considered indicators of an autonomous goal pursuit regardless of whether the goal had been set by a leader or by the follower him-/herself. If we transfer these considerations to the organizational goal setting process, we assume that in order to facilitate followers’ goal pursuit, leaders have to enhance their followers’ evaluation of the goal’s importance and attainability.

### Transformational Leaders as Facilitators of the Goal Pursuit of Employees

Although theoretically the effectiveness of transformational leadership has widely been ascribed to its impact on followers’ perception of organizational goals, empirically this relation experienced far less attention. Those studies which indeed focused on goal attributes found transformational leadership to positively relate to followers’ evaluation of the goal’s specificity and difficulty (Whittington et al., 2004; Bronkhorst et al., 2015), as well as its clarity (Wright et al., 2012). Followers of TLs further rated organizational goals to be more consistent with their own values and interests (Bono and Judge, 2003) and showed a higher agreement with their leaders on strategic goals (Berson and Avolio, 2004). On the team level, transformational leadership was associated with higher levels of team goal commitment (Chi et al., 2011) and a higher congruence with regard to the importance team members attach to the goals (Colbert et al., 2008).

In line with our reasoning on the value of a goal’s importance and attainability in an autonomous goal accomplishment, Latham and Locke (1991) stated that leaders can play a significant role in facilitating their followers’ goal pursuit by convincing them that the goals are both important and attainable. In the present study, we therefore concentrate on these goal attributes and their relation to transformational leadership.

Empirically, transformational leadership has already been related to a goal’s importance (Colbert et al., 2008). This study, though, focused on the degree of goal importance congruence among team members. Finer-grained analyses, however, suggested that rather than the degree of congruence it is an individual’s goal importance perception as such which positively relates to transformational leadership and followers’ job-related attitudes. To substantiate these initial findings and hence theoretical assumptions on the mechanisms of transformational leadership, followers’ individual evaluations of a goal’s importance have to be further examined in the context of these leadership behaviors. Goal clarity, specificity, or difficulty have also been studied with regard to transformational leadership (Wright et al., 2012; Bronkhorst et al., 2015). Besides, this leadership style has been shown to be closely associated with followers’ broader feeling of having the ability to perform successfully (Kark et al., 2003). However, irrespective of the central role it has been assigned theoretically, evidence on the impact of transformational leadership on followers’ perception of a specific goal’s attainability is yet missing. Studies linking transformational leadership and followers’ perception of a goal’s importance and attainability may thus give further evidence-based insides into the process of how TLs transform followers and motivate them to achieve more than expected beyond existing research.

#### Transformational Leadership and Goal Importance

Goal importance refers to the significance an individual assigns to a certain goal and its achievement relative to other work- or non-work-related goals (Hollenbeck and Williams, 1987). It indicates how closely one regulates this goal compared to other goals (Powers, 1978). Goal importance is a significant driver of an individual’s goal commitment (Locke and Latham, 2002), and, as such, aligns one’s feelings and actions to the accomplishment of the specific goal (Hollenbeck and Klein, 1987). As a result, people extend their effort and invest more time even if they face difficulties or obstacles during the goal pursuit. In sum, goal importance is a significant determinant of one’s motivation to achieve certain goals (Bandura, 1997). For this reason, it is of major interest to figure out leadership techniques, which help to increase followers’ perception of an organizational goal’s importance.

In the very beginning, researchers argued that supervisors’ legitimate authority to assign goals or their physical presence was sufficient to create commitment to and raise a goal’s importance (Ronan et al., 1973). Later, Latham and Saari (1979) showed that a supportive leadership style increased the importance attached to goals and that providing a rationale for the goal also functioned as a facilitator (“tell and sell” style; Locke et al., 1988). Moreover, if leaders communicate an inspiring vision they may enhance the attractiveness of attaining a certain goal and accentuate its importance (Berson and Avolio, 2004). Vision articulation, rationales, and a supportive leadership style seem to foster followers’ goal acceptance by making them more likely to see the consequences of goal attainment as rewarding or favorable (Locke and Latham, 2002). In addition, goals gain in importance if followers are involved in the goal setting process. Under this condition, they own the goals agreed upon (Locke and Latham, 2002). Sheldon et al. (2002) developed a goal intervention program, which aimed at increasing one’s sense of ownership. They asked participants to reflect upon the meaningfulness of goals and to consider the core values these goals express (“Own the goal” strategy). Besides, participants were motivated to reflect upon the longer-term goals their current goals serve (“Remember the big picture” strategy). These strategies as well as the leadership attributes, which have been found to strengthen followers’ perception of a goal’s importance, closely match the behaviors TLs use in leading. TLs articulate an ideological vision of an attractive future goal state and frame the work in terms of collectively approved values (Shamir et al., 1993). That way, they provide a meaningful and stimulating rationale for the work to be done but also transform followers’ beliefs and values (Conger and Kanungo, 1998). By aligning followers’ values to the higher-order mission they articulate, TLs create a purpose in work that exceeds beyond extrinsic outcomes (Arnold et al., 2007) and increase the meaningfulness of goal accomplishment (Shamir et al., 1993). Besides strengthening the importance of organizational goals via their alignment to an ideological vision, TLs also foster followers’ sense of ownership by involving them in important organizational decisions. In so doing, TLs delegate responsibilities, are open to followers’ ideas and reasoning, and consider their needs in leading (Avolio et al., 1991).

As TLs present work and especially organizational goals in terms of a higher-order vision and link them to subordinates’ values but also grant subordinates responsibility during the goal pursuit, we assume followers to perceive the goals their TLs set to be more important.

Hypothesis 1: We suggest that the more transformational followers perceive their supervisors to lead, the higher the importance they attach to the organizational goals set by or agreed upon with these leaders.

#### Transformational Leadership and Goal Attainability

Goal setting theory states that for goals to be motivational, they have to be specific and challenging but yet attainable (Locke and Latham, 1990, 2002). Goal attainability indicates how favorable or unfavorable goal strivers perceive external conditions with respect to their goal progress. If an individual perceives a goal to be attainable, he/she has various opportunities to strive toward the goal, has control over the goal striving process, and receives goal-related support from his/her social network (Brunstein, 1993). Accordingly, leaders have three levers to adjust in order to make goals more attainable: opportunities, control, and support.

Social support is an important resource in facilitating employees’ work and enhancing their work attitudes (e.g., Hochwarter et al., 1999; Viswesvaran et al., 1999). In a meta-analysis, Ng and Sorensen (2008) showed that compared to colleagues or the organization as a whole, supervisors are the most valuable source of social support. This value of supervisory support is also acknowledged by the theory of transformational leadership. One of its key components, individualized consideration, includes behaviors such as encouraging followers, acting as their coaches or mentors, and being caring and nurturing (Bass and Avolio, 1994; Conger and Kanungo, 1998). Besides, TLs demonstrate how goals may be attained (Conger, 1999). By providing this kind of social and instrumental support, TLs are likely to positively affect followers’ perception of being able to attain the goals set by their leaders. TLs foster each follower’s personal and professional development (Bass and Avolio, 1994) and promote their growth, independence, and empowerment (Bass, 1985; Kark et al., 2003). To achieve these ends, they use empowering leadership behaviors such as delegating responsibilities and enabling employees to make important decisions, providing resources, and background information about organizational processes, as well as enhancing followers’ capacity to think and question familiar ways of working ultimately raising followers’ self-efficacy beliefs that way (Avolio et al., 1991; Menon, 2001; Dvir et al., 2002; Kark et al., 2003). Self-effective and empowered persons believe in their capability to perform successfully, have a sense of having choice in initiating and regulating actions, and are able to influence outcomes at work (Spreitzer, 1995). As such, these followers ought to feel a higher degree of control with regard to their goal striving. Along with the autonomy they grant, the resources they provide, and the error culture they propagate, the intellectual stimulation TLs practice leads followers to also see and explore new ways of approaching their jobs and completing their tasks (Peng et al., 2016). This motivation to rethink the way they pursue organizational goals likely makes followers aware of new and different opportunities they have in striving toward these goals.

Transformational leaders are hence able to positively impact all three levers leaders may adjust in order to increase followers’ perception of being able to attain their organization’s goals. Therefore, we assume a positive association between transformational leadership and followers’ attainability evaluation of the goals, which had been set by or agreed upon with these leaders.

Hypothesis 2: We suggest that the more transformational followers perceive their supervisors to lead, the higher the attainability they ascribe to the organizational goals set by or agreed upon with these leaders.

#### Transformational Leadership, Goal Attributes, and Followers’ Job Attitudes and Performance

We were not only interested in the question whether TLs are able to facilitate their followers’ goal pursuit but also in showing that this process of motivating and enabling makes a particular contribution to an organization’s functioning. An extant body of meta-analytic evidence shows that TLs substantially influence their subordinates’ job attitudes, motivation, performance, and proactive behavior at work (Fuller et al., 1996; Lowe et al., 1996; Judge and Piccolo, 2004; Wang et al., 2011). Out of the multitude of possible outcomes, we drew on indicators of successful organizational adaptation, as today’s changing work environments and competitive market situation require organizations to easily and quickly adapt to new challenges (Gordon and Yukl, 2004). Specifically, we examined followers’ job satisfaction, organizational commitment, and proactive behavior for indicating an employee’s willingness to accept new challenges in the future (Bateman and Crant, 1993; Cordery et al., 1993; Yousef, 2000).

Previous research also confirmed a clear link between the two goal attributes importance and attainability and followers’ affective job attitudes as well as their performance (e.g., Lee et al., 1991; Maier and Brunstein, 2001; Locke and Latham, 2002). In line with these findings, we assume that TLs facilitate their followers’ goal pursuit process and exert their positive influence on work attitudes and proactive behavior by increasing followers’ perception of the importance and attainability of organizational goals.

Hypothesis 3: We suggest that followers’ evaluations of the organizational goal attributes importance and attainability jointly mediate the relationship between their perception of their leaders’ transformational leadership behavior and (a) their job satisfaction, (b) organizational commitment, and (c) proactive behavior.

## Materials and Methods

### Procedures and Participants

In order to test our hypotheses, we collected data via an online questionnaire at two measurement occasions. At T1, participants were asked to evaluate their leader’s leadership behavior and to list three organizational goals. For each of these goals, participants then indicated its importance and attainability. Job satisfaction, organizational commitment, and proactive behavior were assessed at the second measurement occasion, which was scheduled 4 weeks after the first measures had been taken. We chose this time lag since influences of leadership behavior on employees’ well-being are more likely to be detected within a short than within a long period of time (van Dierendonck et al., 2004). Data sets were matched based on a pre-structured ten-digit code, which participants generated at T1 and T2.

At the beginning and at the end of the first part of the survey, we informed participants that the study consisted of two parts. After completing T1, participants indicated whether they agreed to also respond to the second questionnaire. Those who were inclined to do so were further requested to provide an email address to which the link to the second part was sent by the survey software. In order to ensure anonymity, the survey software had been programmed in a way so that it automatically sent without our assistance a prewritten invitation mail to the second part of the survey to the address participants stated at T1. In the instruction, this procedure was explained in detail. Before we matched the data across measurement occasions and started to analyze them, email addresses were removed from the data set.

Prior to collecting the data, we presented the study to our university’s ethics committee. As it did not deviate from legal regulations or the ethical guidelines of the German Association of Psychology, the ethics committee authorized the study in its final form. Due to the online assessment, we did not personally interact with participants and therefore did not obtain their signed declarations of consent. Yet, we informed them about the study’s content, duration, and aims, and we highlighted that, at any time, participants could abandon the online questionnaire by closing the browser or tab. Participants were assured that incomplete data sets would be deleted and would not be incorporated into our analyses. Moreover, quoting their individual ten-digit code they had developed during the survey, participants were granted the opportunity to still withdraw their data after completing the entire questionnaire.

Participants were recruited in (virtual) business networks and on social media platforms. In sum, 292 employees finished the first part of the questionnaire, but only 144 of them completed its second part. Given the high drop-out rate (50.68%), we compared the responses of those finishing the entire survey with those of participants who did not answer its second part. Analyses did not reveal any systematic drop-out (all *p* > 0.05). Due to missing data across both measurement occasions, we had to exclude 16 participants from the analyses, so that the final sample consisted of 128 followers. Among them, 60.90% were females. The average age was 36.17 years (*SD* = 11.50 years). Participants were employed in a variety of industries (i.e., service companies, retail stores, public services, industrial companies) and had been working for their current organization an average of 8–9 years (*M* = 8.57, *SD* = 8.99). At the time they completed the survey, followers had been collaborating with their current leader for about three and a half years (*M* = 3.52, *SD* = 3.36).

### Measures

#### Listing and Assessment of Organizational Goals

In accordance with prior research (e.g., Maier and Brunstein, 2001), we ideographically assessed organizational goals by asking participants to freely generate and notice up to three work-related goals. Goals were defined as objectives, projects, and plans related to one’s job that were set by or agreed upon with one’s leader. Given the future-orientation of the higher-order vision transformational leaders articulate (Bass, 1985), participants were instructed to focus on those goals they were encouraged to pursue during the following 12 months. After listing these goals, participants indicated the extent to which they perceived each of them to be important and attainable on a five-point response scale ranging from 1 = *not at all* to 5 = *very much*. We computed an overall measure of goal importance and goal attainability by averaging responses across the three goals. A major precondition for aggregating within-person data to the between-person level is sufficient reliability of the aggregate. In order to determine the homogeneity [ICC(1)] and reliability [ICC(2)] of the goal ratings, we calculated intraclass correlation coefficients as suggested by Lüdtke and Trautwein (2007). ICC(1) coefficients were 0.38 for importance and 0.37 for attainability. The corresponding ICC(2) coefficients were 0.65 and 0.64, respectively. ICC(2) is a function of ICC(1) and the number of goals assessed and reliability increases the more goals that are being evaluated. As in the present study only three goals were assessed, intraclass correlation coefficients are within an acceptable range (Lüdtke and Trautwein, 2007).

#### Transformational Leadership

To determine followers’ perceptions of their leaders’ transformational leadership behavior, we used the Transformational Leadership Inventory by Podsakoff et al. (1990); German form: Heinitz and Rowold (2007). With its 22 items, the scale covers the transformational leadership behaviors articulating a vision (“My supervisor paints an interesting picture of the future for our group”), providing an appropriate model (“My supervisor provides a good model for me to follow”), fostering the acceptance of group goals (“My supervisor gets the group to work together for the same goal”), articulating high performance expectations (“My supervisor shows us that he/she expects a lot from us”), providing individualized support (“My supervisor behaves in a manner thoughtful of my personal needs”), and offering intellectual stimulation (“My supervisor challenges me to think about old problems in new ways”). On a response scale ranging from 1 = *never* to 5 = *almost always* followers stated how often their leaders use the behaviors illustrated. The internal consistency of the measure was α = 0.93.

#### Job Satisfaction

Participants’ job satisfaction was measured using the short version of Neuberger and Allerbeck’s (1978) Job Description Form. The unidimensional scale covers one’s satisfaction with seven facets of work (working conditions, tasks, relationship with colleagues, relationship with the supervisor, promotion opportunities, organization and management, and salary). Items were rated on a seven-point Kunin-scale ranging from 1 = *completely dissatisfied* to 7 = *completely satisfied*. Reliability of the scale was 0.82.

#### Organizational Commitment

Organizational commitment was measured with the short version of the Organizational Commitment Questionnaire (Mowday et al., 1979; German form: Maier and Woschée, 2002). Participants were asked to indicate their agreement (ranging from 1 = *strongly disagree* to 5 = *strongly agree*) to nine statements about their identification with and involvement in their organizations (“For me this is the best of all possible organizations for which to work”). Cronbach’s alpha of the scale was 0.91.

#### Proactive Behavior

To assess participants’ proactive behavior, we used the respective subscale of an organizational citizenship behavior questionnaire (Staufenbiel and Hartz, 2000). The scale comprises five items (“I bring in innovative ideas to improve the quality of my department”) which assess an employee’s voluntary behaviors directed at keeping oneself informed about one’s organization, advancing its quality and performance, as well as improving one’s own qualifications. Items were to be answered on a scale ranging from 1 = *strongly disagree* to 5 = *strongly agree* and showed an internal consistency of 0.82.

## Results

Table 1 presents the means, standard deviations, and correlations of the study variables. Hypotheses 1 and 2 assumed a positive association between transformational leadership and followers’ evaluation of the organizational goals that were set by or agreed upon with their leaders. As Table 1 shows, followers’ perception of their leaders’ transformational leadership behavior was indeed positively related to the importance they attach to these goals (*r* = 0.30, *p* < 0.01) and to the attainability they ascribe to them (*r* = 0.23, *p* < 0.01). Hypotheses 1 and 2 are thus supported.

**Table 1:** Descriptive statistics and correlations of the study variables.

Variable	*M*	*SD*	1	2	3	4	5
1. Transformational leadership	3.31	0.63					
2. Goal importance	4.17	0.89	0.30**				
3. Goal attainability	4.05	0.74	0.23**	0.32***			
4. Job satisfaction	4.83	1.01	0.67***	0.34***	0.33***		
5. Organizational commitment	3.45	0.78	0.54***	0.31***	0.23*	0.69***	
6. Proactive behavior	3.72	0.73	0.24**	0.20*	0.29**	0.20*	0.33***

*N = 128. ^∗^p < 0.05, ^∗∗^p < 0.01, ^∗∗∗^p < 0.001.*

**FIGURE 1:**
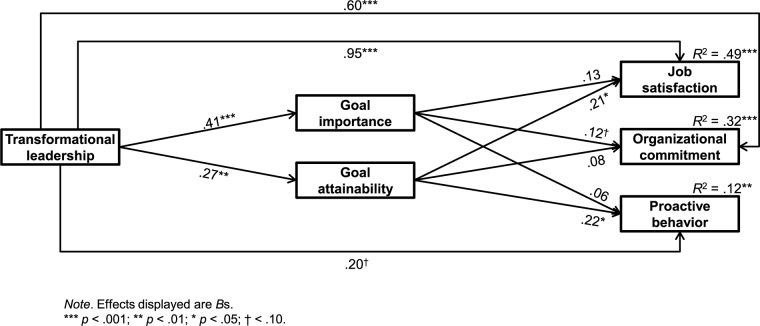
Direct effects of transformational leadership on goal attributes and outcomes as well as of goal attributes on outcomes within the multiple mediation model.

Hypothesis 3 supposed the goal attributes to jointly transmit the effect of transformational leadership on followers’ (a) job satisfaction, (b) organizational commitment, and (c) proactive behavior. To explore this assumption, we tested a multiple mediation model according to Preacher and Hayes (2008) using Hayes’ (2013) PROCESS macro for SPSS. Their approach allows the testing of multiple mediators and multiple outcomes also in smaller samples and accounts for the fact that the sampling distribution of total and indirect effects is commonly not normally distributed (MacKinnon et al., 2004). In order to yield more precise estimates, total and specific indirect effects are bootstrapped and confidence limits for these effects are estimated. In our study, we drew on 95% bias-corrected and accelerated confidence intervals (BCa CI) based on 5,000 bootstrap samples. To test our hypothesis we modeled all variables (transformational leadership as predictor, goal importance and goal attainability as mediators operating in parallel, as well as job satisfaction, organizational commitment, and proactive behavior as outcomes) within a single multiple mediation model. In line with previous meta-analyses (Fuller et al., 1996; Lowe et al., 1996; Judge and Piccolo, 2004; Wang et al., 2011), we found significant total effects of follower-rated transformational leadership on their job satisfaction (*b* = 1.065, BCa CI [0.855, 1.275]), organizational commitment (*b* = 0.671, BCa CI [0.488, 0.853]), and proactive behavior (*b* = 0.282, BCa CI [0.084, 0.480]). For each outcome this effect decreased in size when the goal attributes were considered simultaneously (see the values of the direct effects of transformational leadership on the outcome variables displayed in **Figure 1**). Whereas the direct effect of transformational leadership on job satisfaction and organizational commitment remained significant when controlling for goal attributes suggesting partial mediation, the one on proactive behavior turned out to be only marginally significant under this condition (**Figure 1**). Estimates of the total indirect effect show that, together, both goal attributes mediate the effect of perceived transformational leadership on followers’ job satisfaction (*b* = 0.111, BCa CI [0.028, 0.241]), organizational commitment (*b* = 0.071, BCa CI [0.014, 0.169]), and proactive behavior (*b* = 0.086, BCa CI [0.020, 0.188]). Hypothesis 3 is thus supported. Given that we considered multiple mediators, we could not draw on Preacher and Kelley’s (2011) κ^2^ in determining the size of the indirect effect, but had to rely on the ratio of the indirect effect to the total effect (MacKinnon et al., 1995). One of the disadvantages of this effect size measure is that it may exceed 1 if the indirect effect is bigger than the total effect and may exhibit values below 0 if one of these effects is negative (Hayes, 2013). For job satisfaction, 10.4% of the total effect of transformational leadership was transmitted by the goal attributes, for organizational commitment 10.5% of the total effect resulted from mediation, and in proactive behavior this proportion amounted to 30.4%.

Besides the total indirect effect, PROCESS also estimates the extent to which each mediator transmits the effect of the predictor on the outcome conditional on the presence of the other intervening variables operating in parallel (Preacher and Hayes, 2008). These specific indirect effects give evidence on the relative magnitude of each mediator included in the model. As indicated by the confidence intervals displayed in Table 2, the effect of perceived transformational leadership on job satisfaction and proactive behavior was solely transmitted by followers’ evaluation of the goals’ attainability. With regard to their organizational commitment, we found the effect to be solely mediated by followers’ ratings of the goals’ importance. For this indirect effect, the confidence interval did not include zero. Goal attributes thus seem to be differentially important for the different outcomes.

**Table 2:** Specific indirect effects of transformational leadership on job satisfaction, organizational commitment, and proactive behavior transmitted through the goal attributes goal importance and goal attainability.

	Job satisfaction			Organizational commitment			Proactive behavior		
	*B*	*SE*	95% BCa CI	*B*	*SE*	95% BCa CI	*B*	*SE*	95% BCa CI
Goal importance	0.06	0.04	-0.002; 0.156	0.05	0.03	0.005; 0.140	0.03	0.03	-0.033; 0.104
Goal attainability	0.06	0.04	0.007; 0.153	0.02	0.02	-0.018; 0.083	0.06	0.03	0.016; 0.141

*N = 128.*

## Discussion

The purpose of the present study was to examine the linkage between transformational leadership and followers’ job attitudes as well as their proactive behavior focusing on the goal setting process. We aimed at illustrating that TLs enable followers to autonomously organize their goal pursuit, which we assumed to find expression in higher follower perceptions of the importance and attainability of the goals these leaders set. In line with our assumptions, we indeed found positive relations between follower-rated transformational leadership and their assessment of both goal attributes. TLs articulate an ideological vision and lay emphasis on the meaning of tasks, but also grant followers responsibility and support. Together, these behaviors result in higher levels of identification with and commitment to the organizational goals these leaders set. By demonstrating confidence in their followers’ capability, increasing opportunities for them to significantly affect their work, and providing instrumental and emotional support, TLs lead employees to further perceive these goals to be attainable. Enhancing the importance and attainability of the goals they disseminate, TLs are thus able to facilitate their followers’ organizational goal striving.

In support of our third hypothesis, ratings of the goal attributes mediated the relation between followers’ perceptions of transformational leadership and their job satisfaction, organizational commitment, and proactive behavior. This result supplements earlier findings by Maier and Brunstein (2001) in the domain of personal goals based on which the authors concluded that a sense of commitment to valued goals and the perception of favorable conditions for goal attainment are important requirements for one’s well-being. Our findings suggest that this conclusion also holds when goals are set by a leader instead of followers themselves. Also during the pursuit of assigned goals at work, a goal’s importance and attainability are crucial for success and ultimately for one’s job-related well-being and performance.

Analyses of the specific indirect effects corroborate that goal importance and goal attainability differentially mediated the effect of transformational leadership on the outcomes considered. Whereas transformational leadership and job satisfaction as well as proactive behavior were solely associated via the perception of a goal’s attainability, these leadership behaviors unfolded their impact on followers’ organizational commitment via followers’ perceptions of the goal’s importance only. Concerning followers’ organizational commitment, we think that this mediation can be explained by a spread-out effect in which the appreciation of and identification with a certain vision or goal serves as a proxy for the whole organization. As Mowday et al. (1982) stated, organizational commitment is characterized by “a strong belief in and acceptance of the organization’s goals and values” (p. 27). Therefore, perceiving organizational goals as important is a relevant mechanism in transmitting the effect of transformational leadership on followers’ organizational commitment. Our finding that goal attainability does not significantly mediate this relation might be explained by the fact that employees expect their leaders to facilitate their work in any case (Ng and Sorensen, 2008). Meta-analytic evidence, though, shows followers’ affective commitment to be most affected by perceptions of organizational support (Meyer et al., 2002). As favorable conditions for goal attainment seem to be taken for granted (Ng and Sorensen, 2008) and are thus not perceived as particular support, they probably do not specifically increase followers’ attachment to the organization. With regard to followers’ job satisfaction and proactive behavior, by contrast, goal attainability appeared to be a significant mediator conditional on the presence of goal importance as a second mediator. With regard to one’s satisfaction, this finding is in line with research on personal goals: In this domain, goal attainability has been meta-analytically shown to be associated with an individual’s subjective well-being (e.g., life satisfaction or positive affect); and personal work-related goals were found to more specifically relate to one’s job satisfaction (Klug and Maier, 2015). Unfortunately, the association with a goal’s importance has not been considered within this integrative work. Our findings suggest that in order to be satisfied with one’s job, followers have to be convinced to be able to attain the organizational goals they have been assigned rather than considering these goals to be important. This finding deviates from evidence on the significance of one’s goal commitment within the goal setting theory (for an overview: Locke and Latham, 2002), as well as from evidence on the personal goal model of well-being corroborating that goals need to be both important and attainable in order to increase employees’ job satisfaction (Maier and Brunstein, 2001). In addition, meta-analytic evidence in the field of work design highlights a task’s significance, which is closely associated with an organizational goal’s importance, to be a major correlate of one’s satisfaction with work (Humphrey et al., 2007). As, based on this former research, we would have expected goal importance perceptions to equally mediate the effect of transformational leadership on followers’ job satisfaction, we recommend to reinvestigate the value of followers’ goal importance evaluations in relation to transformational leadership and subordinates satisfaction with work. Also with regard to followers’ proactive behavior, only attainability perceptions mediated the effect of transformational leadership. If employees believe they may affect work outcomes, their willingness to take responsibilities and action is stimulated (Stajkovic and Luthans, 1998). Accordingly, followers who perceive favorable conditions for goal realization are likely to proactively develop these goals and ways to achieve the vision TLs articulate. In previous research, feelings of being able to successfully perform a task have rather been found to moderate the relation between transformational leadership and proactive behavior instead of mediating it (Den Hartog and Belschak, 2012). This earlier work, though, assessed followers’ self-efficacy beliefs, whereas our study focused on the attributes of the goal. Whether TLs exert an identifiable independent influence on both followers’ self-evaluation of their abilities as well as on their perception of the goals’ attributes and – if so – whether these influences operate differently is an important question to answer in future research. Contradicting our assumption, goal importance did not mediate the impact of TLs on followers’ proactive behavior. Maybe, a strong sense of goal importance or commitment may thwart followers’ proactive behavior such that they solely focus on the goal on duty and behaviors directed at attaining this specific goal. In this case, positive effects on followers’ in-role performance are more likely to evolve than effects on their proactive behavior.

### Theoretical Implications and Future Research

Integrating theorizing and research on self-regulated goal pursuit and personal goals with the goal setting of TLs, the present study broadens previous findings on the mechanisms of transformational leadership. Theoretically, it has widely been reasoned that TLs exert their influence on followers’ performance by increasing the importance of organizational goals and boosting followers’ feelings of being able to attain these goals, that way supporting followers’ goal pursuit. Empirical evidence on these deliberations, though, is still scarce. Our results show that TLs facilitate their followers’ goal striving by enhancing their perceptions of the importance and attainability of organizational goals.

The role of TLs within the goal setting process has first been analyzed by Kirkpatrick and Locke (1996). In a laboratory simulation, they found that leaders’ visions affect followers’ performance to the extent that they inspire the setting of specific goals. These researchers, however, investigated quality goals only and the way they assessed goals induced specific (number of errors) rather than vague as well as self-set instead of assigned goals. In the following, Bono and Judge (2003) studied the influence of transformational leadership on followers’ goals among dyads of leaders and followers. They demonstrated that the more transformational supervisors lead, the more self-concordant (i.e., representative for personally held values) are the work goals followers set themselves. Like Kirkpatrick and Locke (1996), also Bono and Judge (2003) focused on followers’ self-generated goals rather than examining the impact of TLs on the organizational goals they set. Other work considered strategic goals disseminated by top management which were assessed and evaluated in qualitative research (Berson and Avolio, 2004), related to an organization’s overall goal (Wright et al., 2012), or did not specifically focus on goals but rather on the way a job is to be done in general (Bronkhorst et al., 2015). The study by Colbert et al. (2008), which also examined a goal’s importance, did not neither refer to goals, which decidedly have been assigned by leaders. They analyzed broader goals, which in a pre-survey have been identified by CEOs to be relevant to the specific industry the research was conducted in (e.g., “Improving customer service” or “Improving the efficiency of internal operations”). Those studies, which indeed investigated organizational goals set by a leader, either viewed goal attributes to moderate the relation between transformational leadership and outcomes (Whittington et al., 2004) or concentrated on the team level evaluation of these attributes (Chi et al., 2011). In our research, we overcome some of these shortcomings: (1) We focused on two decisive goal attributes which have widely been neglected in the study of transformational leadership so far; (2) we concentrated on goals that have been set by leaders – the traditional basis of goal setting theory and one of the main tasks leaders have to complete; and (3) we ideographically assessed organizational goals and followers’ individual evaluations of these goals. Implementing these characteristics, we empirically emphasized goal attributes to be an important mechanism of transformational leadership.

Nevertheless, our findings are just the beginning of systematically bringing together evidence and theorizing on transformational leadership and goals. Future study efforts need to continue this integration. A first step to further intertwine these streams of research is to consider other goal attributes, which have been highlighted to affect the setting of goals (e.g., goal distance, goal orientation, feedback; Locke and Latham, 2002). With regard to followers’ self-efficacy, an important moderator within the goal setting theory, an extensive body of evidence has already been accumulated showing TLs to boost followers’ beliefs in their own (work-related) capabilities (e.g., Pillai and Williams, 2004; Liu et al., 2010; Den Hartog and Belschak, 2012). In addition to considering further mediators and moderators of goal setting, the goal attributes importance and attainability need to be assessed in more detail (e.g., Brunstein, 1993) than we did here.

Moreover, considering the statement by Howell and Shamir (2005) that “leaders and followers both play an active role in shaping their mutual relationships, and therefore shaping organizational outcomes” (p. 108) we argue for a *leader-follower-fit* perspective in future research. The underlying notion of such a perspective is that leaders should tailor their behavior to suit their followers’ needs. Regarding the regulation of one’s goal striving, individuals have certain preferences how to pursue goals (assessment or locomotion regulatory mode) as well as preferences for a desired or undesired end state (promotion or prevention regulatory focus; Higgins, 2000, 2002). The link between transformational leadership and employees’ regulatory *mode* has already been examined empirically (Benjamin and Flynn, 2006). Results demonstrate that followers with more of a locomotion regulatory mode (i.e., desire to move from one state to another) were more affected by TLs than followers with more of an assessment mode (i.e., desire to make comparisons and judgments before acting and appraising performance against standards). This seems to be the case as TLs tend to emphasize movement from state to state. Furthermore, there is evidence that the positive effects of articulating a vision are contingent on follower regulatory *focus*. In two experiments Stam et al. (2010) showed that visions focusing on preventing an undesirable situation lead to better performance than visions focusing on promoting a desirable situation for more prevention-focused followers (who want to avoid failures and fears), while the reverse was true for more promotion-focused followers (who want to reach success and ideals). The fit between followers’ regulatory mode and focus should therefore be further investigated as possible moderator in the interplay of transformational leadership behaviors and followers’ goal striving.

The congruence of leaders’ and followers’ goal appraisals should also be examined. If leaders set their followers goals, an individual redefinition process starts by which followers convert external tasks into internal ones (Hackman, 1970; Hacker, 1982). Employees might be successful in striving for reinterpreted goals, which, in turn, may foster proactive behavior. The question, however, arises whether followers work on the task intended by the leader or whether the redefinition process leads them to work toward goals their leaders never wanted them to pursue. Therefore, research to come should not only assess followers’ evaluation of the goals they have been assigned, but should also consider whether leaders and followers agree upon the content of the goals, which are to be attained.

Just like in everyday life (cf. Austin and Vancouver, 1996), also at work individuals have to simultaneously pursue multiple goals. While acting on the attainment of one goal, employees scan the environment for opportunities to act on the other goals. This may lead to deferrals and reprioritizations of goals of which leaders are unaware. In the field of close relationships, Brunstein et al. (1996) showed that being aware of one’s partners’ goals, significantly influences the association among goal-related support and judgments of marital satisfaction. Only if participants were aware of their partners’ goals, the provision of goal-related support was significantly associated with their partners’ satisfaction. Transferring these findings to the field of leader-follower-interactions, it seems fruitful to explore whether leaders have to know which particular goal their followers actually strive for and how they progress in order to provide the most effective support. As, however, followers and leaders commonly share a more task-oriented relationship than couples, followers might feel controlled instead of empowered under this condition.

### Managerial Implications

Due to its well-established positive impact, transformational leadership has become a prevalent topic in leadership education within business schools throughout the world (Tourish et al., 2010). In small and medium-sized enterprises, however, leaders are rarely recruited from business schools, but rather are promoted into leadership positions based on their technical and professional expertise or the seniority principle. Such leaders often lack knowledge in managing and leading others as well as various skills necessary in successfully facilitating their followers’ goal pursuit. Therefore, they have to be equipped with leadership skills, which are relevant in effectively managing the goal setting process. Previous research has shown that transformational leadership behaviors can be developed in courses or training programs (e.g., Kelloway et al., 2000; Dvir et al., 2002). Such interventions may be tailored to specifically target the dissemination and pursuit of organizational goals. Trainings may start with an examination of the implicit theories of effective leadership and goal setting these leaders have in mind. Via 270- or 360-degree appraisal, they may be given insights into their own leadership behaviors and the way they are perceived by supervisors, colleagues, followers, and – should the occasion arise – customers. These analyses may be used as a starting point to improve the leaders’ behaviors as leaders may deduce a need for development by comparing their ideals and the way they are perceived.

As an important learning goal, leadership trainings need to convey that the manner in which goals are communicated impacts the degree of importance followers attach to these goals. Frese et al. (2003) developed and evaluated an action theory based training to teach participants the inspirational communication of a vision. The training consisted of two components. On the one hand, participants had to develop a vision for their own department and to deliver an enthusiastic and inspiring speech propagating it. Based on feedback, the vision and the speech were constantly improved in further role-plays. On the other hand, participants were taught about the characteristics and the importance of visions. Relevant paralinguistic and content issues of charismatic visions were exemplified and situations in which the speech may be applied were discussed. As evaluation studies of this 1.5 days training module revealed good to excellent effect sizes (Frese et al., 2003), it should be incorporated into broader leadership training programs. Empirical evidence revealed that visions tight to charismatic or transformational leadership among others present an optimistic picture of the future, express confidence that the vision is attainable, or state the importance of followers’ participation (Berson et al., 2001). Contingent reward leaders, by contrast, draw an instrumental vision tight to a specific time frame or linked to extrinsic benefits (Sosik and Dinger, 2007). Thus, in order to be most effective, the particular themes a vision addresses deserve careful consideration within these trainings. Visions contain far-reaching, timeless, and relatively abstract ideas (Berson et al., 2015), whereas goal setting theory found goals to work best if they are specific, challenging and timed (Locke and Latham, 1990). In leading, however, both kinds are important (Latham and Locke, 1991). Berson et al. (2015) reason that the motivational effect of visions vs. goals depends on the characteristics of the specific situation in which they are articulated or assigned: If leaders are socially and spatially proximate to their followers, greater effects result if more specific, time-constrained, and challenging goals are set. If, by contrast, leaders are socially and spatially distant, abstract, far-reaching, and timeless visions are a better means to stimulate followers’ performance. Attributes of the situation and properties of the message a leader delivers, thus need to fit in order to best motivate followers (Berson et al., 2015). Accordingly, apart from learning to develop and articulate inspiring visions to increase the importance of organizational goals, training participants also need to learn about the goal setting theory and how goals need to be formulated and conveyed like it is already done in various transformational leadership trainings (e.g., Barling et al., 1996; Kelloway et al., 2000). In this context, leaders need to learn in which situations best to use either kind of communication strategy.

The communication of more concrete, challenging, and timed goals also helps to increase followers’ trust in being able to achieve the super-ordinate vision (Berson et al., 2015). As such, modules on goal setting also serve in teaching leaders how to increase followers’ perception of an organizational goal’s attainability. Further behaviors, which lead followers to evaluate a goal to be attainable, also need to be developed and practiced in leadership trainings. Accordingly, leaders need to support followers and foster their impression of having control over the goal striving process as well as having several opportunities in achieving a certain goal. In order to increase followers’ perceptions of their control and opportunities, intellectual stimulation is an important leadership behavior. While training leaders, Barling et al. (1996) found this component of transformational leadership to be lowest among those participating in their intervention. To increase intellectually stimulating behaviors, participants were taught about the concept of transformational leadership, role-played these behaviors, and attained four monthly individual booster sessions with the researchers. In addition, leaders were encouraged to discuss new ideas with other training participants themselves in order to practice the behaviors they were meant to increase within their followers. Apart from intellectual stimulation, the information given, the role-plays, as well as the one-to-one coaching sessions also targeted the leaders’ individualized consideration. This behavior is important in fostering followers’ perception of supervisory support. As evidence on the effectiveness of this intervention, followers of those attending the training in sum rated their leaders higher on transformational leadership behaviors than those of a non-participating control group (Barling et al., 1996). Training participants may further be encouraged to see things from their followers’ perspective and to anticipate potential obstacles followers might be confronted with during the goal pursuit. Based on that, leaders may be better able to provide support instrumental in achieving the goals they assign. As, compared to eclectic leadership trainings, transformational leadership trainings resulted in higher ratings of followers’ self-efficacy (Dvir et al., 2002), such trainings should be helpful in increasing followers’ perception of being able to attain the goal their leaders set.

Several months after the initial training, a follow-up session could help to review the implementation of the behavior leaders learned during the training program, to exchange experiences with fellow trainees, and to revise leadership strategies aimed at increasing the importance and attainability of organizational goals. Fellow training participants could provide assistance and feedback on how to transfer the training content into daily work routines and how to deal with obstacles. Such booster sessions aim at maintaining the transfer of training for a longer period of time (Saks and Belcourt, 2006). In sum, transformational leadership trainings have led to modest improvements across the following 2 years (see Bass, 1999).

### Limitations

Despite these contributions, the present study has several limitations. First, our research was solely based on self-report data increasing the possibility of common method and social desirability bias (Podsakoff and Organ, 1986). However, we consciously adopted this approach (see Conway and Lance, 2010) since all of our variables dealt with respondents’ personal cognition and affect. Obviously, respondents themselves are the most reliable and appropriate source of information in this particular case (cf. Chan, 2009). To avoid common method bias, leadership behaviors could have been analyzed as a self-report measure on the part of the leaders. In this study, though, we were interested in the perceptions of followers. Consistent with Walumbwa et al. (2007) we assert that leaders behave differently across situations and individuals or at least are perceived as behaving differently by those affected by these behaviors. Consequently, we actually examined whether differences in the perception of leadership account for variations in followers’ cognition and affect. Although it has been reasoned that the effects of common method variance are overstated (Spector, 2006) and empirical evidence suggests they are leveled out by measurement errors (Lance et al., 2010), we nevertheless collected data at two points in time and ensured participants’ anonymity to reduce possible response biases. Temporal separation of the assessment of predictors and outcomes is one of the procedural remedies suggested by Podsakoff et al. (2012) in order to control for common method biases. By introducing a time lag between these measurements, biases resulting from followers’ desire to appear consistent across responses as well as from demand characteristics related to the specific items may be attenuated (Podsakoff et al., 2012).

The study design with its two temporally separated measurement occasions, however, is associated with a second limitation of the present work: the poor participation of respondents at the second time point and hence the high drop-out rate (cf. Podsakoff et al., 2012). High attrition rates and the associated risk of biased sample selection are particularly common when participants are recruited online and data is collected through the internet at more than one measurement occasion (Kraut et al., 2004). The higher anonymity resulting from the web-based survey method might have caused a decrease in the response rate in our study. Participants did not feel as obliged to fill in the second part of the questionnaire, as they probably would have felt if the data had been collected in cooperation with a specific company. Moreover, we did not offer any kind of incentive, which might have increased the motivation to take part at T2. Nevertheless, we tested for systematic attrition and did not find any differences between respondents and non-respondents.

Although the two-wave study design helps in reducing potential biases resulting from common method variance, it is limited with regard to the examination of mediation effects (Cohen et al., 2003): Based on such a design we may not readily draw rigorous causal inferences (Cole and Maxwell, 2003). Even if we had adopted a sequential design and had added a third time point to measure transformational leadership, the goal attributes, and outcome variables at a distinct time point each, longitudinal mediation would not have been assessed more accurately (Mitchell and Maxwell, 2013). Both designs fail to account for prior levels of the variables and thus for autoregressive effects, which indicate stable individual differences in a certain variable (Preacher, 2015). In order to clarify the causal order of effects, longitudinal designs are needed which assess predictor, mediator, and outcome variables simultaneously at each of various measurement occasions (Cole and Maxwell, 2003). Using such a design, we may rigorously examine the proposed mediating effects, contrast them with alternative causal models, and relate them to concurrent causal influences (Cole and Maxwell, 2003). Given this deficiency in our study design, we have to be careful when interpreting our findings as evidence on the mediation model we assumed, because we may not rule out alternative causal effects. Experimental and training research, however, demonstrated an impact of transformational leadership on followers’ perception of related goal attributes (e.g., Bono and Judge, 2003) just as on the outcomes we considered (e.g., Barling et al., 1996). In the field of personal goals, Maier and Brunstein (2001) provided evidence based on longitudinal data that differences in the interplay between work-related goal commitment and goal attainability reliably predict changes in newcomers’ job satisfaction and job commitment during the first 8 months after organizational entry. In addition, goal effectiveness trainings designed to enhance students’ commitment to goals as well as their goal attainability perceptions improved the effectiveness of the students’ goal striving process and ultimately led to increases in their satisfaction with their studies (Brunstein et al., 2008). Due to their respective designs, these studies allow for strong inferences on causality. The causal effects are in line with the mediation chain we proposed, and therefore reinforce our assumption that transformational leadership affects followers’ perceptions of goal attributes, which in turn exert an influence on their job-related attitudes and proactive behavior. Nonetheless, we recommend future research to further substantiate the impact of transformational leadership on followers’ job satisfaction, commitment, and proactive behavior via goal attributes longitudinally by drawing on cross-lagged panel or latent growth curve models or other currently emerging strategies to model longitudinal mediation (cf. Preacher, 2015).

An additional limitation of our study is that the data was collected in one specific (Western) culture. It is therefore uncertain whether our findings are generalizable across cultures. Given that a cultural influence may especially be assumed with regard to the visionary content transformational leaders convey (House et al., 2004), particularly the impact of TLs on a goal’s importance may vary dependent on the vision theme that is being communicated within a certain culture. In order to yet strengthen the generalizability of our findings, we included a diverse sample representing a broad range of organizations and a variety of industries.

Finally, we cannot rule out that general perceptions of control or support at work might have influenced followers’ ratings of the goal attributes. Future research should consider constructs such as locus of control or decision latitude as well as a supportive organizational culture as influences on followers’ goal attribute perceptions.

## Conclusion

Our study integrates research and theorizing on self-regulatory processes, goal setting, and personal goals in the context of transformational leadership. Although these constructs share certain overlap, they have traditionally been considered from different perspectives. The study empirically supports theoretical assumptions related to the effect of transformational leadership on followers’ goal pursuit showing that TLs influence the extent to which individuals perceive organizational goals as important and attainable. This is remarkable as leading through goals has originally been associated with a task-oriented leadership style according to which leaders set a specific goal, monitor its progress, and allocate rewards. We have learned that TLs exert their impact on followers’ job satisfaction, organizational commitment, and proactive behavior through the goal attributes importance and attainability. Findings suggest that these attributes are decisive in one’s goal striving no matter if a goal is self-set or assigned. However, both goal attributes differentially mediate the effect of transformational leadership. In sum, the present work thus contributes to the fields of leadership as well as goal research and their integration.

## Author Contributions

BS and HK conceptualized the study with careful advice by GM. BS designed the study materials and collected the data. BS and HK processed the data. All authors were concerned with their analysis and interpretation. BS and HK drafted the earlier versions of the manuscript. GM thoroughly commented on these versions inducing further intellectual content. Before submitting the present work, BS substantially revised the manuscript.

## Conflict of Interest Statement

The authors declare that the research was conducted in the absence of any commercial or financial relationships that could be construed as a potential conflict of interest.

## References

[B1] Arnold K. A. Turner N. Barling J. Kelloway E. K. McKee M. C. (2007). Transformational leadership and psychological well-being: the mediating role of meaningful work. *J. Occup. Health Psychol.* 12 193–203. 10.1037/1076-8998.12.3.193 17638487

[B2] Austin J. T. Vancouver J. B. (1996). Goal constructs in psychology: structure, process, and content. *Psychol. Bull.* 120 338–375. 10.1037/0033-2909.120.3.338

[B3] Avolio B. J. Waldman D. A. Yammarino F. J. (1991). Leading in the 1990s: The four I′s of transformational leadership. *J. Eur. Ind. Train.* 15 9–16. 10.1108/03090599110143366

[B4] Bandura A. (1997). *Self-Efficacy.* New York, NY: Freeman.

[B5] Barling J. Weber J. Kelloway E. K. (1996). Effects of transformational leadership training on attitudinal and financial outcomes: a field experiment. *J. Appl. Psychol.* 81 827–832. 10.1037/0021-9010.81.6.827

[B6] Bass B. M. (1985). *Leadership and Performance Beyond Expectations.* New York, NY: Free Press.

[B7] Bass B. M. (1990). *Bass & Stogdill’s Handbook of Leadership.* New York, NY: Free Press.

[B8] Bass B. M. (1999). Two decades of research and development in transformational leadership. *Eur. J. Work Organ. Psychol.* 8 9–32. 10.1080/135943299398410

[B9] Bass B. M. Avolio B. J. (1994). *Improving Organizational Effectiveness Through Transformational Leadership.* Thousand Oaks, CA: Sage.

[B10] Bass B. M. Riggio R. E. (2006). *Transformational Leadership*, 2nd Edn Mahwah, NJ: Erlbaum.

[B11] Bateman T. S. Crant J. M. (1993). The proactive component of organizational behavior: a measure and correlates. *J. Organ. Behav.* 14 103–118. 10.1002/job.4030140202

[B12] Benjamin L. Flynn F. J. (2006). Leadership style and regulatory mode: value from fit? *Organ. Behav. Hum. Decis. Process.* 100 216–230. 10.1016/j.obhdp.2006.01.008

[B13] Berson Y. Avolio B. J. (2004). Transformational leadership and the dissemination of organizational goals: a case study of a telecommunication firm. *Leadersh. Quart.* 15 625–646. 10.1016/j.leaqua.2004.07.003

[B14] Berson Y. Halevy N. Shamir B. Erez M. (2015). Leading from different psychological distances: a construal-level perspective on vision communication, goal setting, and follower motivation. *Leadersh. Quart.* 26 143–155. 10.1016/j.leaqua.2014.07.011

[B15] Berson Y. Shamir B. Avolio B. J. Popper M. (2001). The relationship between vision strength, leadership style, and context. *Leadersh. Quart.* 12 53–73. 10.1016/S1048-9843(01)00064-9

[B16] Bono J. E. Judge T. A. (2003). Self-concordance at work: toward understanding the motivational effects of transformational leaders. *Acad. Manage. J.* 46 554–571. 10.2307/30040649

[B17] Bronkhorst B. Steijn B. Vermeeren B. (2015). Transformational leadership, goal setting, and work motivation: the case of a Dutch municipality. *Rev. Public Pers. Administrat.* 35 124–145. 10.1177/0734371X13515486

[B18] Brunstein J. C. (1993). Personal goals and subjective well-being: a longitudinal study. *J. Pers. Soc. Psychol.* 65 1061–1070. 10.1037/0022-3514.65.5.1061

[B19] Brunstein J. C. Dangelmayer G. Schultheiss O. C. (1996). Personal goals and social support in close relationships: effects on relationship mood and marital satisfaction. *J. Pers. Soc. Psychol.* 71 1006–1019. 10.1037/0022-3514.71.5.1006

[B20] Brunstein J. C. Dargel A. Glaser C. Schmitt C. H. Spörer N. (2008). Persnliche Ziele im Studium: Erprobung einer Intervention zur Steigerung der Zieleffektivität und Zufriedenheit im Studium [Personal goals in college education: testing an intervention to increase students’ goal effectiveness and satisfaction in college]. *Z. Pädagog. Psychol.* 22 177–191. 10.1024/1010-0652.22.34.177

[B21] Brunstein J. C. Schultheiss O. C. Maier G. W. (1999). “The pursuit of personal goals: a motivational approach to well-being and life-adjustment,” in *Action and Development: Theory and Research Through the Life Span*, eds Brandstädter J. Lerner R. M. (Thousand Oaks, CA: Sage), 169–196.

[B22] Chan D. (2009). “So why ask me? Are self-report data really that bad?,” in *Statistical and Methodological Myths and Urban Legends: Received Doctrine, Verity, and Fable in the Organizational and Social Science*, eds Lance C. E. Vandenberg R. J. (Mahwah, NJ: Erlbaum), 311–338.

[B23] Chi N. W. Chung Y. Y. Tsai W. C. (2011). How do happy leaders enhance team success? The mediating roles of transformational leadership, group affective tone and team processes. *J. Appl. Soc. Psychol.* 41 1421–1454. 10.1111/j.1559-1816.2011.00767.x

[B24] Cohen J. Cohen P. West S. G. Aiken L. S. (2003). *Applied Multiple Regression/Correlation Analysis for the Behavioral Sciences*, 3rd Edn Mahwah, NJ: Erlbaum.

[B25] Colbert A. E. Kristof-Brown A. L. Bradley B. H. Barrick M. R. (2008). CEO transformational leadership: the role of goal importance congruence in top management teams. *Acad. Manage. J.* 51 81–96. 10.5465/AMJ.2008.30717744

[B26] Cole D. A. Maxwell S. E. (2003). Testing mediational models with longitudinal data: questions and tips in the use of structural equation modeling. *J. Abnorm. Psychol.* 112 558–577. 10.1037/0021-843X.112.4.558 14674869

[B27] Conger J. A. (1999). Charismatic and transformational leadership in organizations: an insider’s perspective on these developing streams of research. *Leadersh. Quart.* 10 145–179. 10.1016/S1048-9843(99)00012-0

[B28] Conger J. A. Kanungo R. N. (1998). *Charismatic Leadership in Organizations.* Thousand Oaks, CA: Sage.

[B29] Conway J. M. Lance C. E. (2010). What reviewers should expect from authors regarding common method bias in organizational research. *J. Bus. Psychol.* 25 325–334. 10.1007/s10869-010-9181-6

[B30] Cordery J. Sevastos P. Mueller W. Parker S. (1993). Correlates of employee attitudes toward functional flexibility. *Hum. Relat.* 46 705–723. 10.1177/001872679304600602

[B31] Den Hartog D. N. Belschak F. D. (2012). When does transformational leadership enhance employee proactive behavior? The role of autonomy and role breadth self-efficacy. *J. Appl. Psychol.* 97 194–202. 10.1037/a0024903 21842977

[B32] Dvir T. Eden D. Avolio B. J. Shamir B. (2002). Impact of transformational leadership on follower development and performance: a field experiment. *Acad. Manage. J.* 45 735–744. 10.2307/3069307

[B33] Emmons R. A. (1986). Personal strivings: an approach to personality and subjective well-being. *J. Pers. Soc. Psychol.* 51 1058–1068. 10.1037//0022-3514.51.5.1058 7776188

[B34] Frese M. Beimel S. Schoenborn S. (2003). Action training for charismatic leadership: two evaluations of studies of a commercial training module on inspirational communication of a vision. *Pers. Psychol.* 56 671–697. 10.1111/j.1744-6570.2003.tb00754.x

[B35] Fuller J. B. Patterson C. E. P. Hester K. I. M. Stringer D. Y. (1996). A quantitative review of research on charismatic leadership: psychological reports. *Psychol. Rep.* 78 271–287. 10.2466/pr0.1996.78.1.271

[B36] Gordon A. Yukl G. (2004). The future of leadership research: challenges and opportunities. *Ger. J. Hum. Resour. Res.* 18 359–365. 10.1177/239700220401800307

[B37] Hacker W. (1982). “Action control. On the task dependent structure of action-controlling mental representations,” in *Cognitive and Motivational Aspects of Action*, eds Hacker W. Volpert W. Cranach M. (Berlin: Deutscher Verlag der Wissenschaft), 137–149.

[B38] Hackman J. R. (1970). “Tasks and task performance in research on stress,” in *Social and Psychological Factors in Stress*, ed. J. E. McGrath (New York, NY: Holt, Reinhart & Winston), 202–237.

[B39] Hayes A. F. (2013). *Introduction to Mediation, Moderation, and Conditional Process Analysis: A Regression-Based Approach.* New York, NY: The Guilford Press.

[B40] Heckhausen H. Kuhl J. (1985). “From wishes to action: the dead ends and short cuts on the long way to action,” in *Goal-Directed Behavior: Psychological Theory and Research on Action*, eds Frese M. Sabini J. (Hillsdale, NJ: Erlbaum), 134–160.

[B41] Heinitz K. Rowold J. (2007). Gütekriterien einer deutschen Adaptation des Transformational Leadership Inventory (TLI) von Podsakoff [Psychometric properties of a German adaptation of the Transformational Leadership Inventory (TLI) by Podsakoff]. *Z. Arbeits- Organisationspsychol.* 51 1–15. 10.1026/0932-4089.51.1.1

[B42] Higgins E. T. (2000). Making a good decision: value from fit. *Am. Psychol.* 55 1217–1230. 10.1037/0003-066X.55.11.1217 11280936

[B43] Higgins E. T. (2002). How self-regulation creates distinct values: the case of promotion and prevention decision making. *J. Consum. Psychol.* 12:177 10.1207/S15327663JCP1203_01

[B44] Hochwarter W. A. Perrewé P. L. Ferris G. R. Brymer R. A. (1999). Job satisfaction and performance: the moderating effects of value attainment and affective disposition. *J. Vocat. Behav.* 54 296–313. 10.1006/jvbe.1998.1659

[B45] Hollenbeck J. R. Klein H. J. (1987). Goal commitment and the goal-setting process: problems, prospects, and proposals for future research. *J. Appl. Psychol.* 72 212–220. 10.1037/0021-9010.72.2.212

[B46] Hollenbeck J. R. Williams C. R. (1987). Goal importance, self-focus and the goal-setting process. *J. Appl. Psychol.* 72 204–211. 10.1037/0021-9010.72.2.204

[B47] House R. J. Hanges P. J. Javidan M. Dorfman P. W. Gupta V. (2004). *Culture, Leadership, and Organizations: The GLOBE Study of 62 Societies.* Thousand Oaks, CA: Sage.

[B48] Howell J. M. Shamir B. (2005). The role of followers in the charismatic leadership process: relationships and their consequences. *Acad. Manage. Rev.* 30 96–112. 10.2307/20159097

[B49] Humphrey S. E. Nahrgang J. D. Morgeson F. P. (2007). Integrating motivational, social, and contextual work design features: a meta-analytic summary and theoretical extension of the work design literature. *J. Appl. Psychol.* 92 1332–1356. 10.1037/0021-9010.92.5.1332 17845089

[B50] Judge T. A. Piccolo R. F. (2004). Transformational and transactional leadership: a meta-analytic test of their relative validity. *J. Appl. Psychol.* 89 755–768. 10.1037/0021-9010.89.5.755 15506858

[B51] Kark R. Shamir B. Chen G. (2003). The two faces of transformational leadership: empowerment and dependency. *J. Appl. Psychol.* 88 246–255. 10.1037/0021-9010.88.2.246 12731708

[B52] Karoly P. (1993). Mechanisms of self regulation: a systems view. *Annu. Rev. Psychol.* 44 23–52. 10.1146/annurev.ps.44.020193.000323

[B53] Kelloway E. K. Barling J. Helleur J. (2000). Enhancing transformational leadership: the roles of training and feedback. *Leadersh. Organ. Dev. J.* 21 145–149. 10.1108/01437730010325022 27345643

[B54] Kirkpatrick S. A. Locke E. A. (1996). Direct and indirect effects of three core charismatic leadership components on performance and attitudes. *J. Appl. Psychol.* 81 36–51. 10.1037/0021-9010.81.1.36

[B55] Klug H. J. P. Maier G. W. (2015). Linking goal progress and subjective well-being: a meta-analysis. *J. Happin. Stud.* 16 37–65. 10.1007/s10902-013-9493-0

[B56] Kraut R. Olson J. Banaji M. Bruckman A. Cohen J. Couper M. (2004). Psychological research online: report of board of scientific affairs’ advisory group on the conduct of research on the internet. *Am. Psychol.* 59 105–117. 10.1037/0003-066X.59.2.105 14992637

[B57] Lance C. E. Dawson B. Birkelbach D. Hoffman B. J. (2010). Method effects, measurement error, and substantive conclusions. *Organ. Res. Methods* 13 435–455. 10.1177/1094428109352528

[B58] Latham G. P. Locke E. A. (1991). Self-regulation through goal setting. *Organ. Behav. Hum. Decis. Process.* 50 212–247. 10.1016/0749-5978(91)90021-K

[B59] Latham G. P. Saari L. M. (1979). Importance of supportive relationships in goal settings. *J. Appl. Psychol.* 64 151–156. 10.1037//0021-9010.64.2.151

[B60] Lee C. Bobko P. Earley P. C. Locke E. A. (1991). An empirical analysis of a goal setting questionnaire. *J. Organ. Behav.* 12 467–482. 10.1002/job.4030120602

[B61] Liu J. Siu O.-L. Shi K. (2010). Transformational leadership and employee well-being: the mediating role of trust in the leader and self-efficacy. *Appl. Psychol. Int. Rev.* 59 454–479. 10.1111/j.1464-0597.2009.00407.x

[B62] Locke E. A. Latham G. P. (1990). *A Theory of Goal Setting and Task Performance.* Eaglewood Cliffs, NJ: Prentice Hall.

[B63] Locke E. A. Latham G. P. (2002). Building a practically useful theory of goal setting and task motivation: a 35-year odyssey. *Am. Psychol.* 57 705–717. 10.1037/0003-066X.57.9.705 12237980

[B64] Locke E. A. Latham G. P. Erez M. (1988). The determinants of goal commitment. *Acad. Manage. Rev.* 13 23–39. 10.5465/AMR.1988.4306771

[B65] Lowe K. B. Kroeck K. G. Sivasubramaniam N. (1996). Effectiveness correlates of transformational and transactional leadership: a meta-analytic review of the MLQ literature. *Leadersh. Quart.* 7 385–425. 10.1016/S1048-9843(96)90027-2

[B66] Lüdtke O. Trautwein U. (2007). Aggregating to the between-person level in idiographic research designs: personal goal research as an example of the need to distinguish between reliability and homogeneity. *J. Res. Pers.* 41 230–238. 10.1016/j.jrp.2006.03.005

[B67] MacKinnon D. P. Lockwood C. M. Williams J. (2004). Confidence limits for the indirect effect: distribution of the product and resampling methods. *Multivar. Behav. Res.* 39 99–128. 10.1207/s15327906mbr3901_4 20157642 PMC2821115

[B68] MacKinnon D. P. Warsi G. Dwyer J. H. (1995). A simulation study of mediated effect measures. *Multivar. Behav. Res.* 30 41–62. 10.1207/s15327906mbr3001_3 20157641 PMC2821114

[B69] Maier G. W. Brunstein J. C. (2001). The role of personal work goals in newcomers’ job satisfaction and organizational commitment: a longitudinal analysis. *J. Appl. Psychol.* 86 1034–1042. 10.1037/0021-9010.86.5.1034 11596797

[B70] Maier G. W. Woschée R. M. (2002). Die affektive Bindung an das Unternehmen: Psychometrische Überprüfung einer deutschsprachigen Fassung des Organizational Commitment Questionnaire (OCQ) von Porter und Smith (1970) [Affective commitment to an organization: psychometric examination of a German version of the Organizational Commitment Questionnaire (OCQ) of Porter and Smith (1970)]. *Z. Arbeits- Organisationspsychol*. 46 126–136. 10.1026//0932-4089.46.3.126

[B71] Menon S. T. (2001). Employee empowerment: an integrative psychological approach. *Appl. Psychol. Int. Rev.* 50 153–180. 10.1111/1464-0597.00052

[B72] Meyer J. P. Stanley D. J. Herscovitch L. Topolnytsky L. (2002). Affective, continuance, and normative commitment to the organization: a meta-analysis of antecedents, correlates, and consequences. *J. Vocat. Behav.* 61 20–52. 10.1006/jvbe.2001.1842

[B73] Mitchell M. A. Maxwell S. E. (2013). A comparison of the cross-sectional and sequential designs when assessing longitudinal mediation. *Multivar. Behav. Res.* 48 301–339. 10.1080/00273171.2013.784696 26741846

[B74] Mowday R. T. Steers R. M. Porter L. W. (1979). The measurement of organizational commitment. *J. Vocat. Behav.* 14 224–247. 10.1016/0001-8791(79)90072-1

[B75] Mowday R. T. Steers R. M. Porter L. W. (1982). *Employee-Organizational Linkages: The Psychology of Commitment, Turnover and Absenteeism.* New York, NY: Academic Press.

[B76] Neuberger O. Allerbeck M. (1978). *Messung und Analyse von Arbeitszufriedenheit [Measurement and Analysis of Job Satisfaction].* Bern: Huber.

[B77] Ng T. W. H. Sorensen K. L. (2008). Toward a further understanding of the relationships between perceptions of support and work attitudes: a meta-analysis. *Group Organ. Manage.* 33 243–268. 10.1177/1059601107313307

[B78] Oettingen G. Pak H. Schnetter K. (2001). Self-regulation of goal setting: turning free fantasies about the future into binding goals. *J. Pers. Soc. Psychol.* 80 736–753. 10.1037/0022-3514.80.5.736 11374746

[B79] Peng A. C. Lin H.-E. Schaubroeck J. Mcdonough E. F. Hu B. Zhang A. (2016). CEO intellectual stimulation and employee work meaningfulness: the moderating role of organizational context. *Group Organ. Manage.* 41 203–231. 10.1177/1059601115592982

[B80] Pillai R. Williams E. A. (2004). Transformational leadership, self-efficacy, group cohesiveness, commitment and performance. *J. Organ. Change Manage.* 17 144–159. 10.1108/09534810410530584

[B81] Podsakoff P. M. MacKenzie S. B. Moorman R. H. Fetter R. (1990). Transformational leader behaviors and their effects on followers’ trust in leader, satisfaction, and organizational citizenship behaviors. *Leadersh. Quart.* 1 107–142. 10.1016/1048-9843(90)90009-7

[B82] Podsakoff P. M. MacKenzie S. B. Podsakoff N. P. (2012). Sources of method bias in social science research and recommendations on how to control it. *Annu. Rev. Psychol.* 63 539–569. 10.1146/annurev-psych-120710-100452 21838546

[B83] Podsakoff P. M. Organ D. W. (1986). Self-reports in organizational research: problems and prospects. *J. Manage.* 12 531–544. 10.1177/014920638601200408 8452065

[B84] Powers W. T. (1978). Quantitative analysis of purposive systems: some spadework at the foundations of scientific psychology. *Psychol. Rev.* 85 417–435. 10.1037/0033-295X.85.5.417

[B85] Preacher K. J. (2015). Advances in mediation analysis: a survey and synthesis of new developments. *Annu. Rev. Psychol.* 66 825–852. 10.1146/annurev-psych-010814-015258 25148853

[B86] Preacher K. J. Hayes A. F. (2008). Asymptotic and resampling strategies for assessing and comparing indirect effects in multiple mediator models. *Behav. Res. Methods* 40 879–891. 10.3758/BRM.40.3.879 18697684

[B87] Preacher K. J. Kelley K. (2011). Effect size measures for mediation models: quantitative strategies for communicating indirect effects. *Psychol. Methods* 16 93–115. 10.1037/a0022658 21500915

[B88] Ronan W. W. Latham G. P. Kinne S. B. (1973). Effects of goal setting and supervision on worker behavior in an industrial situation. *J. Appl. Psychol.* 58 302–307. 10.1037/h0036303

[B89] Saks A. M. Belcourt M. (2006). An investigation of training activities and transfer of training in organizations. *Hum. Resour. Manage.* 45 629–648. 10.1002/hrm.20135 15239319

[B90] Shamir B. House R. J. Arthur M. B. (1993). The motivational effects of charismatic leadership: a self-concept based theory. *Organ. Sci.* 4 577–594. 10.1287/orsc.4.4.577

[B91] Sheldon K. M. Kasser T. Smith K. Share T. (2002). Personal goals and psychological growth: testing an intervention to enhance goal attainment and personality integration. *J. Pers.* 70 5–31. 10.1111/1467-6494.00176 11908535

[B92] Sosik J. J. Dinger S. L. (2007). Relationships between leadership style and vision content: the moderating role of need for approval, self-monitoring, and need for social power. *Leadersh. Quart.* 18 134–153. 10.1016/j.leaqua.2007.01.004

[B93] Spector P. E. (2006). Method variance in organizational research. *Organ. Res. Methods* 9 221–232. 10.1177/1094428105284955

[B94] Spreitzer G. M. (1995). Psychological empowerment in the workplace: dimensions, measurement, and validation. *Acad. Manage. J.* 38 1442–1465. 10.2307/256865

[B95] Stajkovic A. D. Luthans F. (1998). Self-efficacy and work-related performance: a meta-analysis. *Psychol. Bull.* 124 240–261. 10.1037/0033-2909.124.2.240

[B96] Stam D. A. van Knippenberg D. Wisse B. (2010). The role of regulatory fit in visionary leadership. *J. Organ. Behav.* 31 499–518. 10.1002/job.624

[B97] Staufenbiel T. Hartz C. (2000). Organizational Citizenship Behavior: Entwicklung und erste Validierung eines Meßinstruments [Organizational citizenship behavior: development and validation of a measurement instrument]. *Diagnostica* 46 73–83. 10.1026//0012-1924.46.2.73

[B98] Tett R. P. Guterman H. A. Bleier A. Murphy P. J. (2000). Development and content validation of a “hyperdimensional” taxonomy of managerial competence. *Hum. Perform.* 13 205–251. 10.1207/S15327043HUP1303_1

[B99] Tourish D. Craig R. Amernic J. (2010). Transformational leadership education and agency perspectives in business school pedagogy: a marriage of inconvenience? *Br. J. Manage.* 21:s40 10.1111/j.1467-8551.2009.00682.x

[B100] van Dierendonck D. Haynes C. Borrill C. Stride C. (2004). Leadership behavior and subordinate well-being. *J. Occup. Health Psychol.* 9 165–175. 10.1037/1076-8998.9.2.165 15053715

[B101] Vancouver J. B. (2000). “Self-regulation in organizational settings: a tale of two paradigms,” in *Handbook of Self-Regulation*, eds Boekaerts M. Pintrich P. Zeidner M. (San Diego, CA: Academic Press), 303–341. 10.1016/B978-012109890-2/50039-1

[B102] Viswesvaran C. Sanchez J. I. Fisher J. (1999). The role of social support in the process of work stress: a meta-analysis. *J. Vocat. Behav.* 54 314–334. 10.1006/jvbe.1998.1661 9325800

[B103] Walumbwa F. O. Lawler J. J. Avolio B. J. (2007). Leadership, individual differences, and work-related attitudes: a cross-culture investigation. *Appl. Psychol.* 56 212–230. 10.1111/j.1464-0597.2006.00241.x

[B104] Wang G. Oh I. Courtright S. H. Colbert A. E. (2011). Transformational leadership and performance across criteria and levels: a meta-analytic review of 25 years of research. *Group Organ. Manage.* 36 223–270. 10.1177/1059601111401017

[B105] Whittington J. L. Goodwin V. L. Murray B. (2004). Transformational leadership, goal difficulty, and job design: independent and interactive effects on employee outcomes. *Leadersh. Quart.* 15 593–606. 10.1016/j.leaqua.2004.07.001

[B106] Wright B. E. Moynihan D. P. Pandey S. K. (2012). Pulling the levers: transformational leadership, public service motivation, and mission valence. *Public Administrat. Rev.* 72 206–215. 10.1111/j.1540-6210.2011.02496.x

[B107] Yousef D. A. (2000). Organizational commitment and job satisfaction as predictors of attitudes toward organizational change in a non-western setting. *Pers. Rev.* 29 567–592. 10.1108/00483480010296401

